# Supplementary material for: Integrated omics networks reveal the temporal signaling events of brassinosteroid response in Arabidopsis
Source: Nat Commun. 2021 Oct 6;12:5858. doi: 10.1038/s41467-021-26165-3 (PMC8494934; doi:10.1038/s41467-021-26165-3)
Supplement: Supplementary file 1 — Supplementary Information [file 41467_2021_26165_MOESM1_ESM.pdf]

Supplementary Information

Supplementary Methods

The Spatiotemporal Clustering and Inference of Omics Networks (SC-ION) pipeline is based on several previously described computational methods. We describe those methods here.

Regression Tree Pipeline for Spatial, Temporal, and Replicate Data (RTP-STAR)[1]

RTP-STAR was developed to infer dynamic Gene Regulatory Networks (GRNs) from steady-state, spatial transcriptomic data. It is a MATLAB-based pipeline and is available on GitHub: https://github.com/nmclark2/RTP-STAR. There are three main components of this pipeline. The first is clustering co-expressed transcripts as it has been shown that clustering improves the overall precision of the inferred network[2]. The second is network inference using a regression-tree based method named GEne Network Inference with Ensemble of trees 3 (GENIE3)[3]. This network inference is performed on each individual cluster to generate one network per cluster. The individual networks can then be merged using their hub genes (genes with the highest out degree). The third and final component is the inference of the sign of the edge (activation or repression) using the first order Markov method[2]. This third component is the only part of RTP-STAR that requires time course data. When implementing SC-ION, we kept the clustering of co-expressed gene products, but allowed the choice of either Dynamic Time Warping (DTW)[4] or Independent Component Analysis (ICA)[5] clustering depending on the type of data. We also allowed for the option of network inference without the clustering step, or for the user to input their own pre-determined list of clusters. We continue to infer GRNs using GENIE3. However, we have not implemented the first order Markov method for edge sign determination into SC-ION at this time. Finally, we have implemented SC-ION in the R language.

GEne Network Inference with Ensemble of trees 3 (GENIE3)[3]

GENIE3 is a regression tree-based network inference pipeline that is implemented in many computational languages, including MATLAB and R. Our SC-ION pipeline uses the R implementation of GENIE3. Regression tree inference methods treat network inference as a feature selection problem where the expression of a gene of interest can be modeled as a function of the expression of all of the other genes in the network. The best fitting model, or network, will minimize the error between the predicted gene expression value and the experimentally measured value. Analytically computing every

possible model would be computationally expensive: thus, GENIE3 uses some methods to reduce the computational cost while maintaining the precision of the network inference. The first is the use of bootstrapping for feature selection: rather than considering all of the genes, GENIE3 considers a randomly selected subset. The second is the use of tree-based ensemble methods for network generation: by generating many networks from these bootstrapped gene sets, one can average the networks to obtain the best-fitting network (due to the Law of Large Numbers). This results in a network inference method that is robust and precise. GENIE3 has been shown to perform well in challenges using simulated and experimental data (such as the DREAM challenges) and has been used widely throughout different biological fields.

SC-ION implements a modified version of GENIE3 based on previous results. The first modification is the addition of edge trimming. One of the key assumptions of GENIE3 is that the resulting networks are connected: that is, all of the genes can be reached by all of the other genes when following the network edges. However, previous work has showed that trimming the number of edges can increase the precision of biological networks[6]. RTP-STAR chose to trim the edges in GENIE3 based on the proportion of regulators in the network: the more regulators, the more edges that are kept[1]. We use this same edge trimming in our SC-ION pipeline.

The second modification is the addition of a second data matrix to allow for the integration of different data types. GENIE3 uses one data matrix for both the regulators and targets of the network. While this works well in experiments that only use one type of data, such as transcriptomic studies, it does not allow for one to consider using different data types for regulators and targets (e.g. using protein abundance for regulators and transcript abundance for targets). Thus, we use a modified version of GENIE3 that allows for the incorporation of both a regulator and a target data matrix[7].

Dynamic Time Warping (DTW)[4]

DTW refers to a class of algorithms that compare two series of values to each other. The rationale behind DTW is to stretch or compress one data series to best fit another data series through constructing a data transformation function called a warping function. There are many applications of DTW methods, one of which is clustering time series data. This is done through calculating the alignment of one time series to another. One way of calculating the alignment is by calculating the average distance

between the time series based on Euclidean distance. Another method is examining the warping function to determine how many time points in one series map to the same time point in the other series (e.g. t_1=1 maps to t_2=1, t_1=2 maps to t_2=2, etc.). SC-ION uses this second method to cluster time series data via the R package *dtw*. For each pair of genes, the warping function between their time series data is calculated. SC-ION then computes the proportion of time points which map to each other via the warping function. If this proportion exceeds a certain threshold (typically at least half of the points must align to each other, although we allow the user to choose a different threshold), those genes are clustered together. One advantage of DTW is that it non-random: the warping function between two expression profiles will always be the same, so if the clustering threshold is not changed, the same genes will always cluster together. One disadvantage of DTW is that it is quite slow since the warping function must be individually calculated for each pair of genes. Additionally, DTW works best on sequential data, such as time series data, but does not perform as well on non-sequential data, such as spatial data.

Independent Component Analysis (ICA)[5]

ICA assumes that a set of gene expression measurements, such as those from a time series experiment, can be modelled as a linear combination of statistically independent components. By determining these independent components, ICA reduces the dimensionality of the dataset while still retaining the key information necessary for clustering. SC-ION uses ICAclust, which first converts the data into its independent components and then clusters the components using hierarchical clustering. While ICA was first developed and tested on biological time series data, it was also shown to perform well on simulated data that did not necessarily come from a sequential dataset. ICAclust is computationally fast and can cluster thousands of genes in just a few minutes. This is due to the dimensionality reduction of the dataset into its independent components. Additionally, the use of hierarchical clustering results in non-random clusters. Unlike DTW, ICA works well on both time series and spatial data. However, ICA does have some disadvantages. First, ICA attempts to determine the optimal number of clusters using Mojena’s criterion, which is based on the first stage of the hierarchical clustering dendrogram. While this criterion works well for a small number of genes, it can become difficult to determine the correct number of clusters for larger datasets based on the dendrogram alone. Second, the algorithm cannot re-evaluate genes once they are clustered. For example, if two genes are clustered together at the beginning of the

algorithm, they can never be separated, even if later in the algorithm the data suggest a different clustering configuration. This combination of Mojena's criterion and hierarchical clustering can lead to many individual clusters with similar data expression patterns that potentially should be merged.

We provide both clustering methods in SC-ION based on the robustness of their results on different types of biological data. In some situations, one method might perform better than the other in terms of computational time or cluster precision. However, in general, we find that using both methods on the same dataset leads to similar clustering results and conclusions.

Supplementary Figures

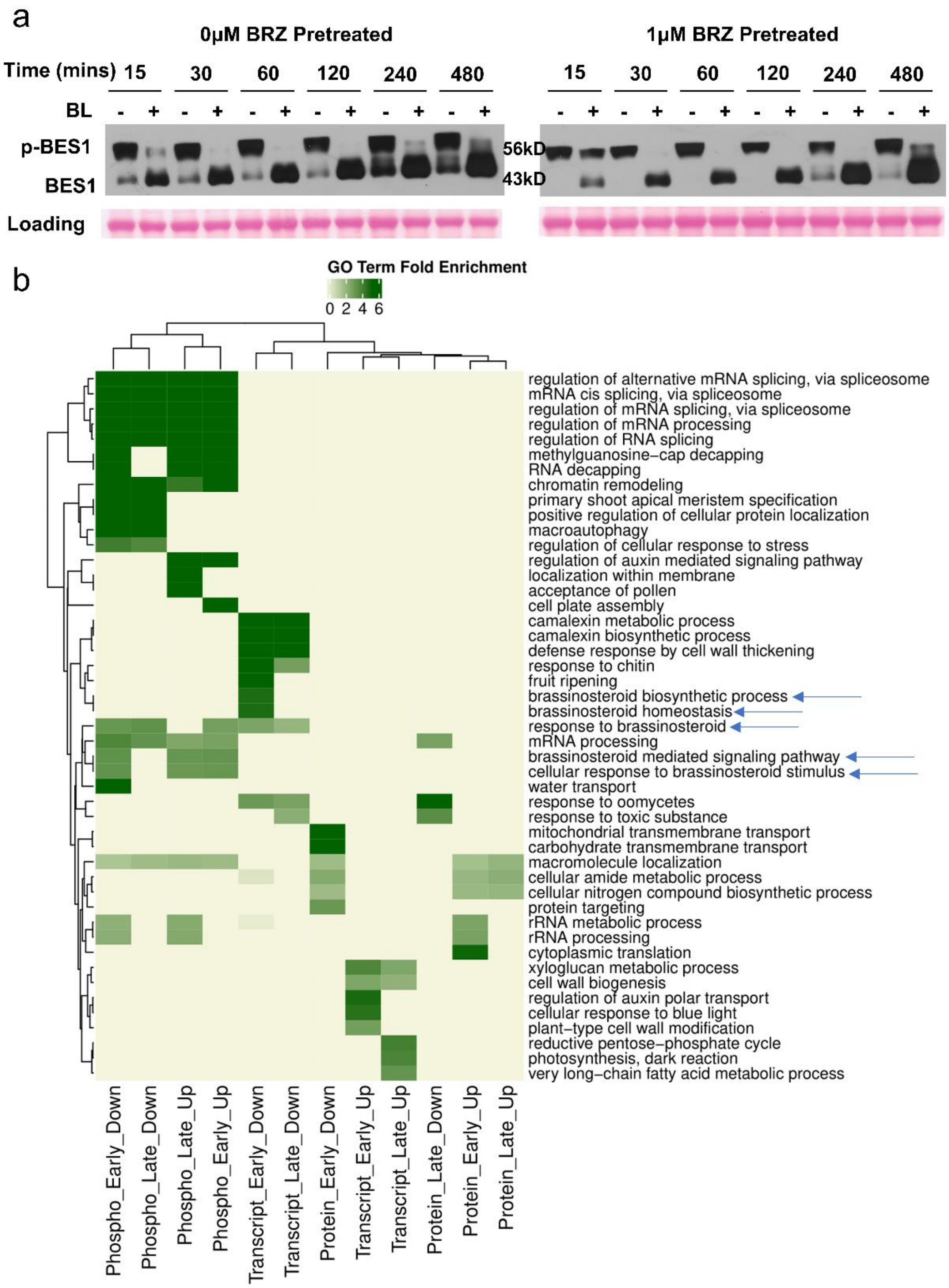

Supplementary Figure 1. Effect of BL treatment on transcript, non-modified protein, and phosphorylated protein levels. (a) Western blot of phosphorylated (p-BES1) and non-modified BES1 protein after BL treatment. (left) No BRZ pre-treatment. (right) BRZ pre-treatment. Blot was repeated n=2 times with similar results. (b) Heatmap of GO terms enriched for different differentially expressed gene-products (transcript, protein, phospho) at different times (early, 1 hour or earlier after BL treatment) (late, after 2 hours of BL treatment). Blue arrows denote BR-related GO terms.

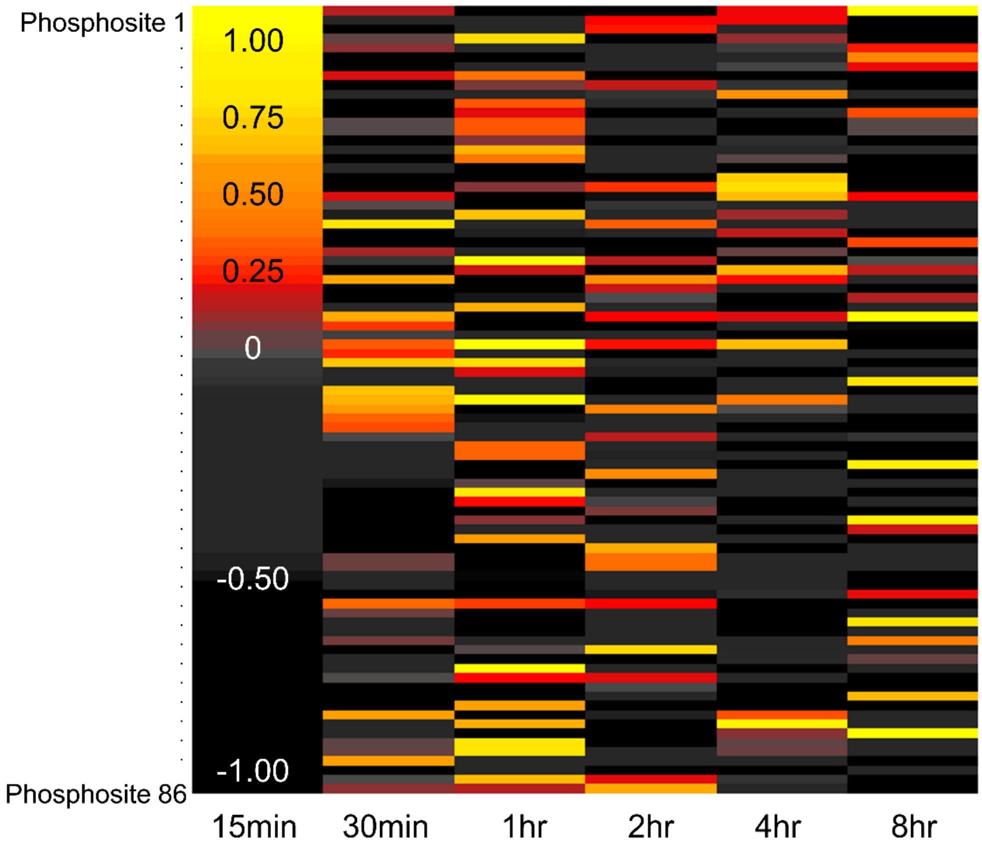

Supplementary Figure 2. Correlation between non-modified protein abundance and phosphosite abundance for kinases DE in response to BR. We identified 86 phosphosites as p-loop activation domains that were DE in response to BR. We then correlated the protein abundance with the phosphosite intensity values for these 86 sites, which are shown in this heatmap. Yellow represents the highest positive correlation, orange is moderate correlation, and red to black is low to no correlation. Phosphosites are rows, time points are columns.

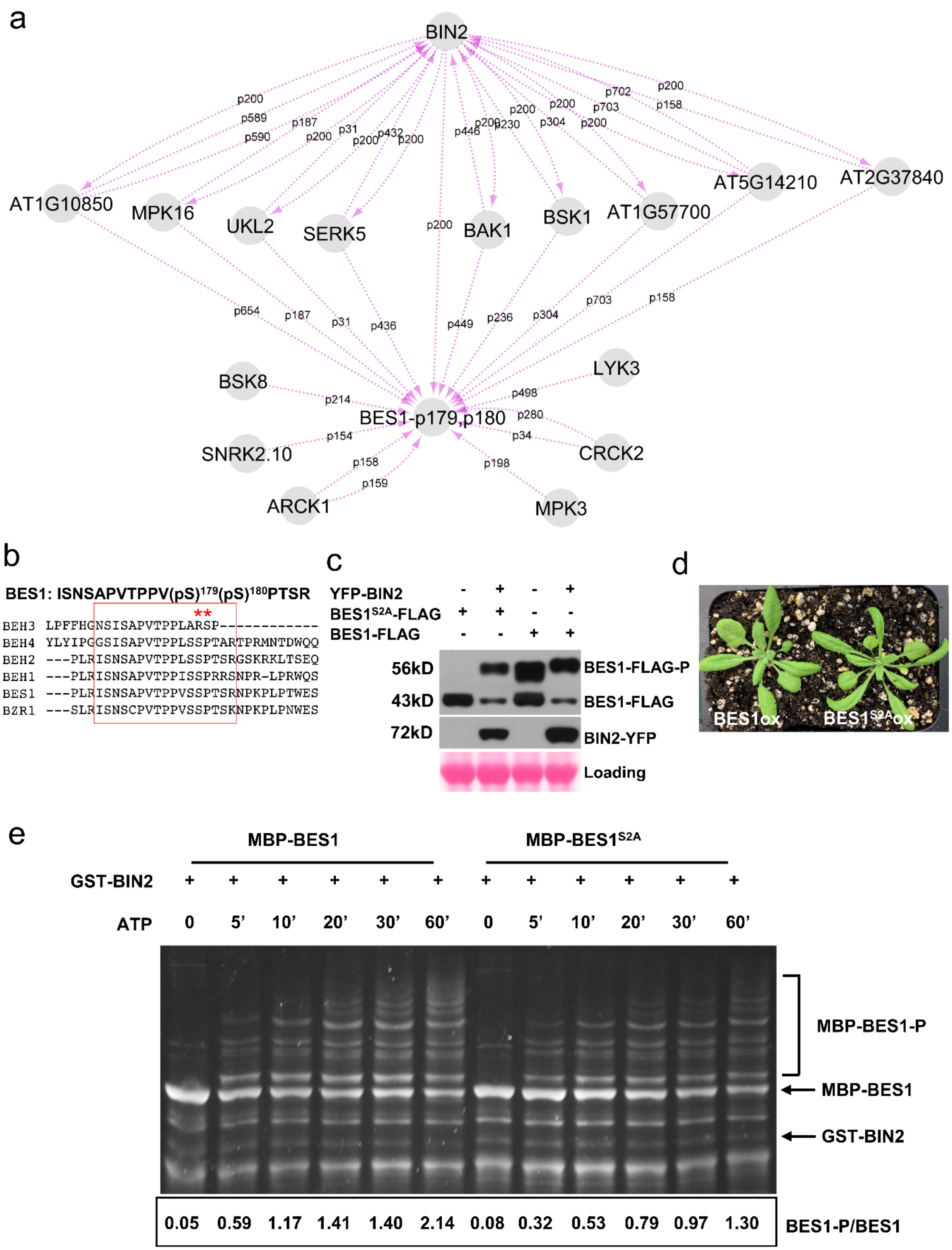

Supplementary Figure 3. Network prediction and experimental validation that S179, and S180 on BES1 are BIN2-phosphorylation sites (a) Kinase-signaling network prediction for the doubly-phosphorylated

form of BES1, which is phosphorylated on sites 179 and 180. Edge labels represent the phosphosite used to predict the regulation. (b) Conservation of S179 and S180 in BES1 with its homologs. Protein alignment was performed using Clustal Omega (https://www.ebi.ac.uk/Tools/msa/clustalo/) . (c) Transient expression of *BES1-FLAG* and *BES1^{S2A}-FLAG* in *Nicotiana benthamiana* leaves, with or without YFP-BIN2. Blot was repeated n=3 times with similar results. (d) Growth phenotype of 3-week-old T1 transgenic plants of BES1-FLAG (BES1ox, left) and BES1^{S2A}-FLAG (BES1^{S2A}ox, right). n=20 out of 26 BES1^{S2A}-FLAG plants showed the BR-gain-of-function growth phenotype. Each T1 plant represents an independent transgenic line. (e) In-vitro kinase assay measuring BIN2 phosphorylation of BES1 and BES1^{S2A}. Numbers at the bottom are the BES1-P/BES1 ratio for each sample. Blot was repeated n=3 times with similar results.

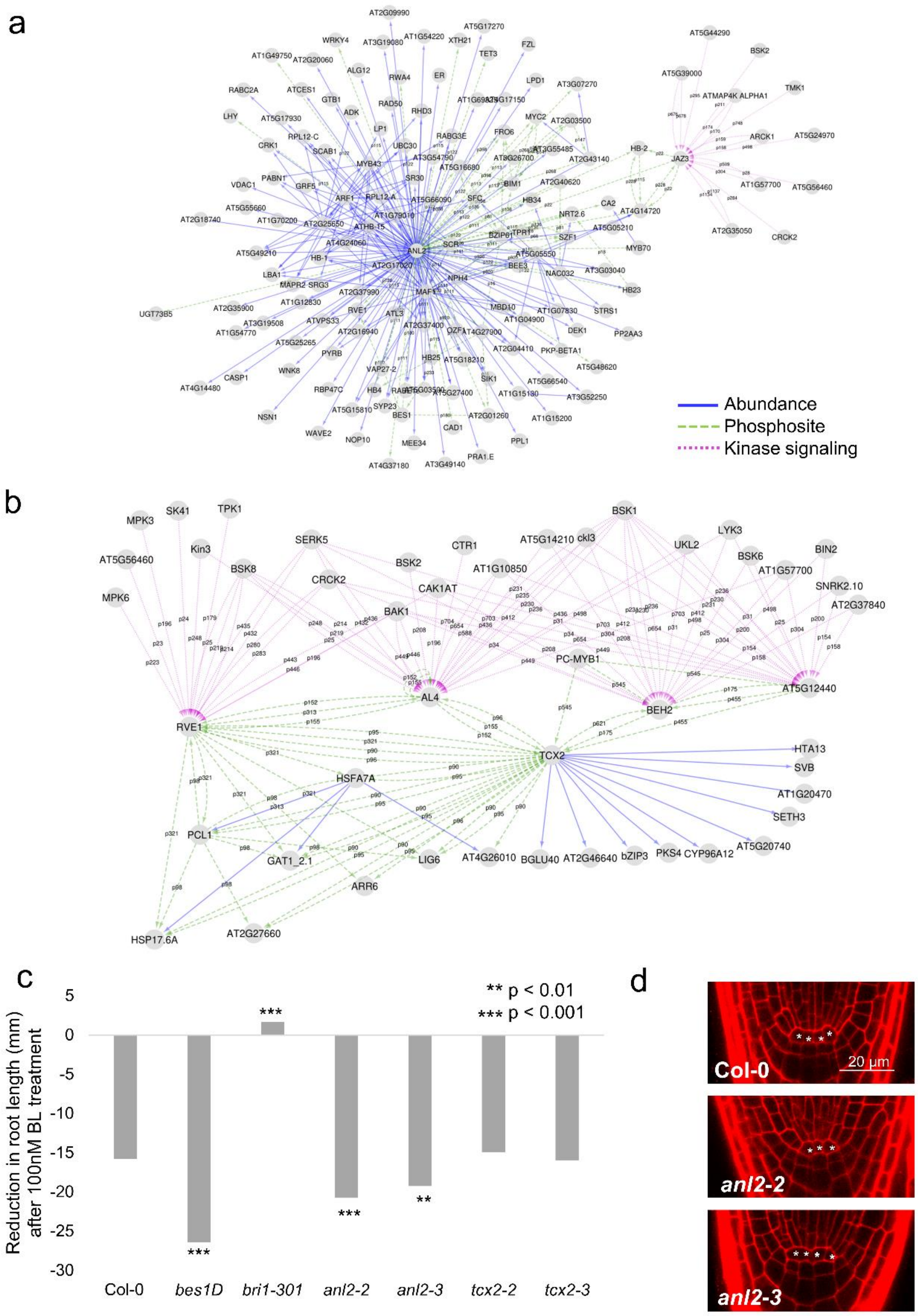

Supplementary Figure 4. ANL2 is a BR-responsive TF. (A,B) First-neighbor subnetworks of TCX2 (a) and ANL2 (b). TF-abundance, blue solid; TF-phosphosite, green dashed; kinase signaling, purple dotted. For kinase-signaling, edge labels represent the phosphosite used to predict the regulation. (c) Response of *tcx2* and *anl2* mutant roots to 100 nM BL treatment. ** denotes $p < 0.01$, *** denotes $p < 0.001$, generalized mixed linear model. (d) Representative images of 5-day-old Col-0, *anl2-2* and *anl2-3* roots (n=12 per genotype). * denotes QC cells.

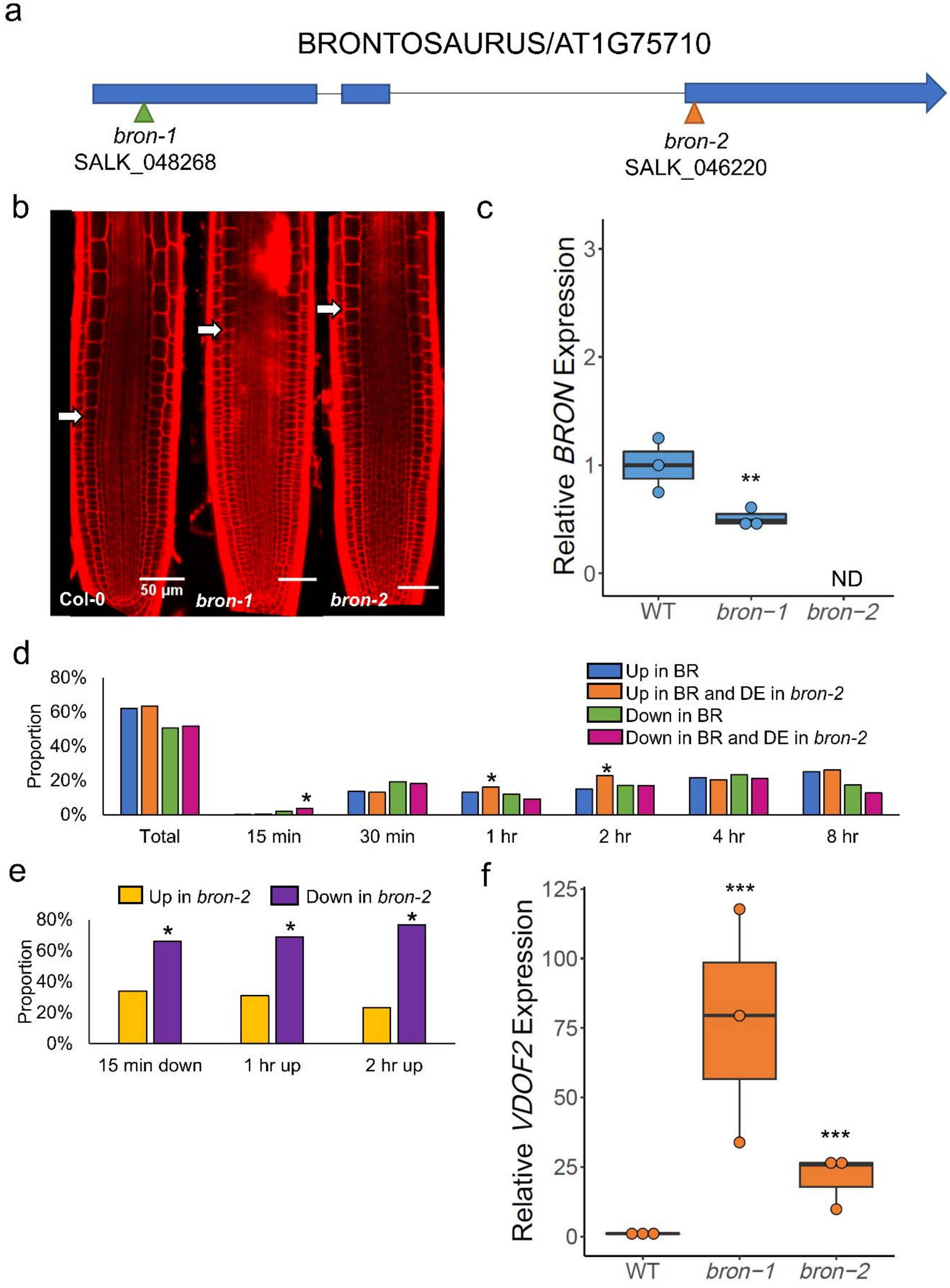

Supplementary Figure 5. Interplay between BRONTOSAURUS expression and BR signaling. (a) Gene model of BRONTOSAURS showing locations of TDNA insertions. (b) Representative images of 5-day-old root meristems of Col-0 (n=22), *bron-1* (n=18) and *bron-2* (n=18) mutants. Arrows mark the end of the

meristem and the beginning of the transition zone. (c) RT-qPCR of *BRON* in *bron* mutant alleles. Reported values are $2^{-\Delta\Delta CT}$. Center line represents median; box bounds represent 25th and 75th percentiles; whiskers represent minimum and maximum. Colored dots represent the average of two technical replicates for each biological replicate (n=3). ND: not detected. ** denotes p<0.01 using a two-tailed *z*-test compared to mean and standard deviation of WT. (d) Proportion of genes induced (blue) or repressed (green) by BR that are also differentially expressed in the *bron-2* mutant (orange and magenta, respectively). * denotes significant enrichment with p<0.05 using hypergeometric test for enrichment. (e) Proportion of genes repressed by BR at 15 minutes or induced by BR at 1 or 2 hours that are up (yellow) or down (purple) in the *bron-2* mutant. * denotes p<0.05, Chi-squared test with likelihood ratio. (f) RT-qPCR of *VDOF2* in *bron* mutant alleles. Reported values are $2^{-\Delta\Delta CT}$. Center line represents median; box bounds represent 25th and 75th percentiles; whiskers represent minimum and maximum. Colored dots represent the average of two technical replicates for each biological replicate (n=3). *** denotes p<0.0001 using *z*-test compared to mean and standard deviation of WT. For all statistical tests, no multiple testing correction was performed.

a

CYCLINs DE in *bron-2*

ID	Symbol	Direction
AT4G34160	CYCD3;1	Up
AT3G60550	CYCU2;2/CYCP3;2	Dn
AT2G44740	CYCU4;1/CYCP4;1	Up
AT5G61650	CYCU4;3/CYCP4;2	Up

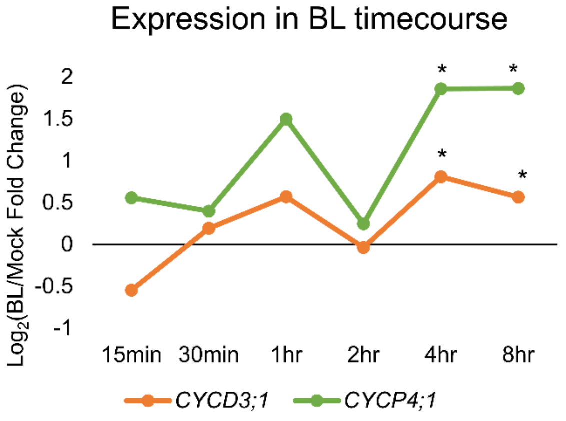

b

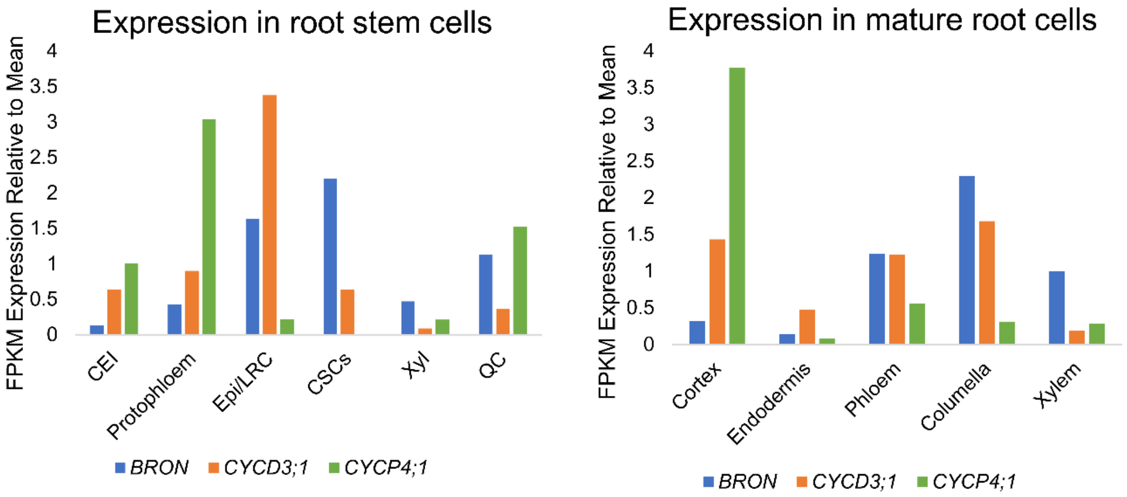

Supplementary Figure 6. Expression of *CYCLIN* genes in BL time course and *bron-2* mutant roots. (a) (left) Cyclins differentially expressed in the *bron-2* mutant. (right) BL/Mock transcript fold change of *CYCD3;1* (orange) and *CYCP4;1* (green) in the BL time course. * denotes the gene is differentially expressed in response to BL at that time point. (b) Transcript expression of *BRON* (blue), *CYCD3;1* (orange), and *CYCP4;1* (green) in root stem cell populations (left, from[1]) and mature root tissues (right, from[8]). CEI – Cortex Endodermis Initials; Epi/LRC – Epidermis/Lateral Root Cap Initials; CSCs – Columella Stem Cells; Xyl – Xylem initials; QC – Quiescent Center. Reported expression is FPKM in each cell type normalized to the mean FPKM across cell types (relative expression).

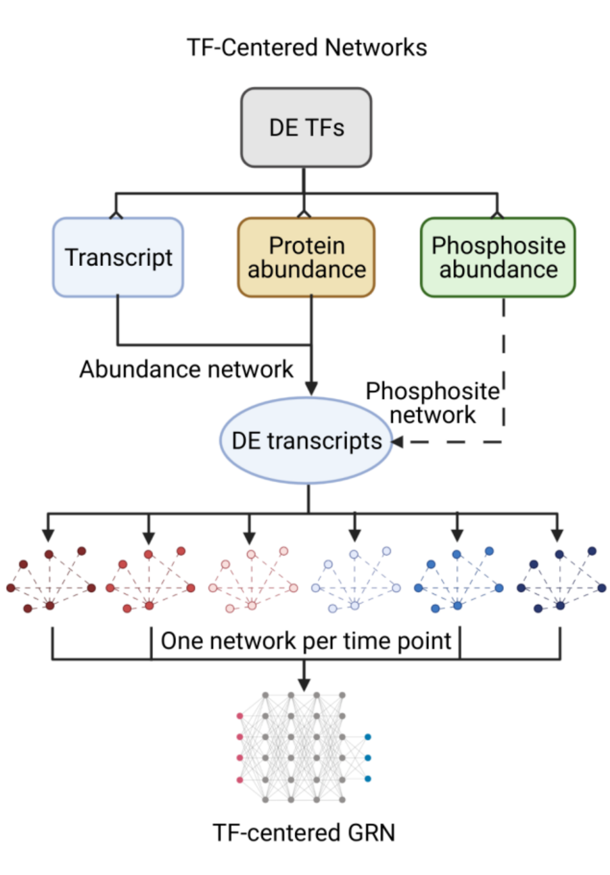

Supplementary Figure 7. Workflow of network inference. For the TF-centered networks (left), DE TFs were used as the regulators of the network. TFs were detected in our time course at transcript, protein abundance, and/or phosphosite abundance levels. In the abundance network, protein abundance (or transcript abundance when the cognate protein was not quantified) was used for the TFs. In the phosphosite network, the phosphosite abundance was used for the TFs. In both networks, transcript expression was used for the target genes. One network was inferred for the DE genes at each time point, and the networks were then combined in a union to form the TF-centered GRN. For the kinase-signaling networks (right), DE kinase p-loop phosphosites were used as the regulators of the network. Pearson and Spearman correlation values were calculated between the p-loop sites and all DE phosphosites across all time points. Edges were kept if they had a Pearson correlation ≥ 0.5 or a Spearman correlation ≥ 0.6. These edges form the kinase signaling GRN.

Supplementary Tables

Supplementary Table 1. ChIP validation of predicted direct targets of BES1 from the integrated omics network.

target	ChIP
AT1G13920	NO
AT1G16610	NO
AT1G20970	NO
AT1G23380	NO
AT1G23760	NO
AT1G32360	NO
AT1G32700	YES
AT1G62300	YES
AT1G71520	NO
AT2G29480	NO
AT2G30530	YES
AT2G32730	NO
AT2G33380	NO
AT2G45660	YES
AT2G47210	NO
AT3G24020	NO
AT3G26180	NO
AT3G29075	NO
AT3G44750	NO
AT3G49620	YES
AT3G51140	NO
AT3G57570	NO
AT5G10200	NO
AT5G43830	NO
AT1G16390	NO
AT1G69030	YES
AT2G15020	NO
AT2G18160	YES
AT2G29300	NO
AT2G32560	YES
AT2G34180	NO
AT2G44430	NO
AT3G14810	NO
AT3G53620	NO
AT4G03390	NO
AT5G03520	YES
AT5G47640	YES
AT5G65613	NO
AT1G01060	NO

AT1G05300	YES
AT1G09770	NO
AT1G12740	NO
AT1G13260	YES
AT1G17840	NO
AT1G30320	YES
AT1G54120	YES
AT1G58100	YES
AT1G66200	NO
AT1G69570	YES
AT1G73390	NO
AT1G76500	YES
AT2G01260	NO
AT2G23320	YES
AT2G28930	YES
AT2G37950	NO
AT3G07570	NO
AT3G25890	NO
AT3G47500	NO
AT3G47620	YES
AT3G55980	YES
AT4G00730	YES
AT4G01810	NO
AT4G18880	YES
AT4G35750	NO
AT4G36970	NO
AT5G03470	NO
AT5G14050	NO
AT5G15130	NO
AT5G17300	NO
AT5G28300	YES
AT5G38600	NO
AT5G39660	NO
AT5G41190	NO
AT5G42920	NO
AT5G49230	NO
AT5G51440	NO
AT1G49750	NO
AT2G32540	NO
AT3G02800	NO
AT3G19450	NO
AT3G48430	NO
AT1G01540	YES

AT1G04240	YES
AT1G04430	NO
AT1G06350	NO
AT1G07370	NO
AT1G10550	YES
AT1G12460	NO
AT1G15010	NO
AT1G19968	NO
AT1G29790	NO
AT1G30690	YES
AT1G34370	NO
AT1G49380	NO
AT1G55330	YES
AT1G62300	YES
AT1G62520	YES
AT1G67750	NO
AT1G72430	YES
AT1G76160	YES
AT2G19580	YES
AT2G20760	YES
AT2G26250	NO
AT2G32690	NO
AT2G33330	NO
AT2G41820	NO
AT2G41940	YES
AT2G46710	YES
AT2G47070	YES
AT3G01970	NO
AT3G02170	YES
AT3G03850	YES
AT3G05840	YES
AT3G06770	NO
AT3G07010	YES
AT3G16910	NO
AT3G23805	NO
AT3G27650	NO
AT3G28130	NO
AT3G43720	NO
AT3G46550	YES
AT3G46940	NO
AT3G57780	NO
AT3G58790	NO
AT4G00330	NO

AT4G09160	NO
AT4G14360	NO
AT4G14440	NO
AT4G14548	YES
AT4G24275	NO
AT4G29240	NO
AT4G30800	YES
AT4G34490	NO
AT4G36240	NO
AT5G01090	YES
AT5G07870	NO
AT5G16000	NO
AT5G16590	NO
AT5G18150	YES
AT5G18690	NO
AT5G19290	NO
AT5G20130	NO
AT5G23860	YES
AT5G36260	NO
AT5G41400	YES
AT5G44670	NO
AT5G46730	NO
AT5G49170	NO
AT5G51460	NO
AT5G52280	YES
AT5G54250	NO
AT5G55960	YES
AT5G56010	NO
AT5G56860	YES
AT5G62140	NO
AT5G63180	YES
AT5G64770	NO

Supplementary Table 2. BR phenotyping on mutants of interest. Values are root length in mm. A generalized mixed linear model with penalized quasi-likelihood was used to determine the genotype x treatment interaction *p*-values. No multiple testing correction was performed.

**Line**	**BL0 Mean**	**BL100 Mean**	**Change in root length**	**p-val**	**Number of mock seedlings**	**Number of BR-treated seedlings**
WT	32.84	17.04	-15.80		73	73
bes1D	31.02	4.59	-26.43	6.37E-06	28	28
bri1-301	26.36	28.06	1.69	5.71E-10	27	29
anl2-2	38.51	17.75	-20.76	3.21E-03	40	34
anl2-3	31.60	12.35	-19.25	1.33E-02	51	37
tcx2-2	36.77	21.87	-14.90	5.71E-01	47	44
tcx2-3	30.70	14.71	-15.99	3.43E-01	46	42
bron-1	40.31	9.57	-30.73	2.37E-19	48	39
bron-2	31.12	10.46	-20.65	7.44E-04	43	54

Supplementary Table 3. RT-qPCR results. Values are $2^{-\Delta\Delta C_T}$. SE: standard error. ND: not detected. NT: not tested. For *tmk1-1*, three biological replicates were assayed, but *BRON* was detected in only two replicates. A two-tailed *z*-test was used to compare each sample to the mean and standard deviation of the expression of the target gene in WT. No multiple testing correction was performed.

**Line**	**Target**	**Mean**	**SE**	**p-val**	**biological replicates**
WT	*BRON*	1.000	0.150		3
bron-1	*BRON*	0.509	0.052	0.0011	3
bron-2	*BRON*	ND	0.000	NT	3
kin10	*BRON*	0.998	0.162	0.9889	3
mpk6-4	*BRON*	1.933	0.625	<0.0001	3
tmk1-1	*BRON*	0.607	0.381	0.0264	3 (detected in 2)
bak1-4	*BRON*	1.345	0.485	0.0217	3
map4ka1	*BRON*	0.179	0.088	<0.0001	3
vdof2	*BRON*	0.123	0.080	<0.0001	3
WT	*VDOF2*	1.000	0.337		3
bron-1	*VDOF2*	76.995	24.248	<0.0001	3
bron-2	*VDOF2*	20.913	5.565	<0.0001	3

Supplementary Table 4. GO enrichment analysis of genes DE in *bron-2* and in BL time course.
Green squares denote that term is enriched in that group of genes.

**GO term**	Dn BR 15min & Up *bron-2*	Dn BR 15min & Dn *bron-2*	Up BR 1hr & Dn *bron-2*	Up BR 2hr & Dn *bron-2*
toxin metabolic process (GO:0009404)	■			
defense response (GO:0006952)	■	■		
response to biotic stimulus (GO:0009607)	■	■		
response to abiotic stimulus (GO:0009628)	■	■	■	■
response to fungus (GO:0009620)	■	■		
response to stress (GO:0006950)	■	■		
response to oxygen levels (GO:0070482)		■		
response to salicylic acid (GO:0009751)		■		
response to wounding (GO:0009611)		■		
immune response (GO:0006955)		■		
response to ethylene (GO:0009723)		■		
response to water deprivation (GO:0009414)		■		
response to hormone (GO:0009725)		■		
cell wall organization or biogenesis (GO:0071554)			■	
response to karrikin (GO:0080167)			■	■
response to temperature stimulus (GO:0009266)			■	■
flavonoid biosynthetic process (GO:0009813)				■
response to heat (GO:0009408)				■
response to UV (GO:0009411)				■
response to stimulus (GO:0050896)				■

Supplementary Table 5. TMT labeling strategy for quantitative proteomics. Ref: Pooled reference. Sample names are formatted as Treatment_Time_Replicate.

**BR1**	TMT label	Sample	Channel
97.5% labeling efficiency	126	Ref	1
	127N	Mock_15min_1	2
	127C	Mock_15min_2	3
	128N	Mock_15min_3	4
	128C	Mock_15min_4	5
	129N	BR_15min_1	6
	129C	BR_15min_2	7
	130N	BR_15min_3	8
	130C	BR_15min_4	9
	131	Ref	10
**BR2**	TMT label	Sample	Channel
97.7% labeling efficiency	126	Ref	1
	127N	Mock_30min_1	2
	127C	Mock_30min_2	3
	128N	Mock_30min_3	4
	128C	Mock_30min_4	5
	129N	BR_30min_1	6
	129C	BR_30min_2	7
	130N	BR_30min_3	8
	130C	BR_30min_4	9
	131	Ref	10
**BR3**	TMT label	Sample	Channel
97.4% labeling efficiency	126	Ref	1
	127N	Mock_1hr_1	2
	127C	Mock_1hr_2	3
	128N	Mock_1hr_3	4
	128C	Mock_1hr_4	5
	129N	BR_1hr_1	6
	129C	BR_1hr_2	7
	130N	BR_1hr_3	8
	130C	BR_1hr_4	9
	131	Ref	10
**BR4**	TMT label	Sample	Channel
98.7% labeling efficiency	126	Ref	1

	127N	Mock_2hr_1	2
	127C	Mock_2hr_2	3
	128N	Mock_2hr_3	4
	128C	Mock_2hr_4	5
	129N	BR_2hr_1	6
	129C	BR_2hr_2	7
	130N	BR_2hr_3	8
	130C	BR_2hr_4	9
	131	Ref	10
**BR5**	TMT label	Sample	Channel
98.5% labeling efficiency	126	Ref	1
	127N	Mock_4hr_1	2
	127C	Mock_4hr_2	3
	128N	Mock_4hr_3	4
	128C	Mock_4hr_4	5
	129N	BR_4hr_1	6
	129C	BR_4hr_2	7
	130N	BR_4hr_3	8
	130C	BR_4hr_4	9
	131	Ref	10
**BR6**	TMT label	Sample	Channel
94.7% labeling efficiency	126	Ref	1
	127N	Mock_8hr_1	2
	127C	Mock_8hr_2	3
	128N	Mock_8hr_3	4
	128C	Mock_8hr_4	5
	129N	BR_8hr_1	6
	129C	BR_8hr_2	7
	130N	BR_8hr_3	8
	130C	BR_8hr_4	9
	131	Ref	10

Supplementary Table 6. Primers used in this study

**Primer**	**Sequence**
BES1^{S2A} Forward Primer	CAGTGGCTGCTCCAACTTCTAGAAACC
BES1^{S2A} Reverse Primer	GTTGGAGCAGCCACTGGTGGA GTGACAGG
BES1 Full Length Forward Primer	CGCGGTACCATGACGTCTGACGGAGCAACGTCG
BES1 Full Length Reverse Primer	CGCGGTACCACTATGAGCTTTACCATTTCCAA
BRON Forward Primer	AGGGTTTGTGCTTGTTCCCA
BRON Reverse Primer	GTTTCGACCCGAATCCTCCG
VDOF2 Forward Primer	CCATTTGAGAGCCTCCTCAAACTT
VDOF2 Reverse Primer	TTGCCATGAACCACCAGATGC
AT4G34270 Forward Primer	GTGAAAACTGTTGGAGAGAAGCAA
AT4G34270 Reverse Primer	TCAACTGGATACCCTTTCGCA

Supplemental References

1. Clark, N. M. *et al.* Stem-cell-ubiquitous genes spatiotemporally coordinate division through regulation of stem-cell-specific gene networks. *Nat. Commun.* **10**, 5574 (2019).
2. Balaguer, M. A. de L. *et al.* Predicting gene regulatory networks by combining spatial and temporal gene expression data in Arabidopsis root stem cells. *Proc. Natl. Acad. Sci.* **114**, E7632–E7640 (2017).
3. Huynh-Thu, V. A., Irrthum, A., Wehenkel, L. & Geurts, P. Inferring Regulatory Networks from Expression Data Using Tree-Based Methods. *PLOS ONE* **5**, e12776 (2010).
4. Giorgino, T. Computing and Visualizing Dynamic Time Warping Alignments in *R* : The **dtw** Package. *J. Stat. Softw.* **31**, (2009).
5. Nascimento, M. *et al.* Independent Component Analysis (ICA) based-clustering of temporal RNA-seq data. *PLOS ONE* **12**, e0181195 (2017).
6. Shibata, M. *et al.* GTL1 and DF1 regulate root hair growth through transcriptional repression of ROOT HAIR DEFECTIVE 6-LIKE 4 in Arabidopsis. *Development* **145**, (2018).
7. Walley, J. W. *et al.* Integration of omic networks in a developmental atlas of maize. *Science* **353**, 814–818 (2016).
8. Li, S., Yamada, M., Han, X., Ohler, U. & Benfey, P. N. High-Resolution Expression Map of the Arabidopsis Root Reveals Alternative Splicing and lincRNA Regulation. *Dev. Cell* **39**, 508–522 (2016).
