# Supplementary material for: Integrated omics networks reveal the temporal signaling events of brassinosteroid response in Arabidopsis
Source: Nat Commun. 2021 Oct 6;12:5858. doi: 10.1038/s41467-021-26165-3 (PMC8494934; doi:10.1038/s41467-021-26165-3)
Supplement: Supplementary file 3 — Description of Additional Supplementary Files [file 41467_2021_26165_MOESM3_ESM.pdf]

Description of Additional Supplementary Files

Supplementary Data 1: Transcripts differentially expressed in response to BR

Supplementary Data 2: Protein groups differentially expressed in response to BR

Supplementary Data 3: Phosphorylation sites differentially expressed in response to BR

Supplementary Data 4: GO analysis on DE transcripts, protein groups, and phosphosites

Supplementary Data 5: Inferred kinase-signaling and TF-centered networks

Supplementary Data 6: Network Motif Analysis on TF-centered networks

Supplementary Data 7: Transcripts differentially expressed in the bron-2 mutant

Supplementary Data 8: Overlap of transcripts DE in the bron-2 mutant and the BR timecourse

Supplementary Data 9: GO analysis on transcripts DE in the bron-2 mutant and the BR timecourse
