# Supplementary material for: Integrated omics networks reveal the temporal signaling events of brassinosteroid response in Arabidopsis
Source: Nat Commun. 2021 Oct 6;12:5858. doi: 10.1038/s41467-021-26165-3 (PMC8494934; doi:10.1038/s41467-021-26165-3)
Supplement: Supplementary file 13 — Reporting Summary [file 41467_2021_26165_MOESM13_ESM.pdf]

nature research

Corresponding author(s): Justin W Walley

Last updated by author(s): Aug 24, 2021

Reporting Summary

Nature Research wishes to improve the reproducibility of the work that we publish. This form provides structure for consistency and transparency in reporting. For further information on Nature Research policies, see our Editorial Policies and the Editorial Policy Checklist.

Statistics

For all statistical analyses, confirm that the following items are present in the figure legend, table legend, main text, or Methods section.

n/a	Confirmed	
☐	☒	The exact sample size (*n*) for each experimental group/condition, given as a discrete number and unit of measurement
☐	☒	A statement on whether measurements were taken from distinct samples or whether the same sample was measured repeatedly
☐	☒	The statistical test(s) used AND whether they are one- or two-sided<br>*Only common tests should be described solely by name; describe more complex techniques in the Methods section.*
☒	☐	A description of all covariates tested
☐	☒	A description of any assumptions or corrections, such as tests of normality and adjustment for multiple comparisons
☐	☒	A full description of the statistical parameters including central tendency (e.g. means) or other basic estimates (e.g. regression coefficient) AND variation (e.g. standard deviation) or associated estimates of uncertainty (e.g. confidence intervals)
☐	☒	For null hypothesis testing, the test statistic (e.g. *F*, *t*, *r*) with confidence intervals, effect sizes, degrees of freedom and *P* value noted<br>*Give P values as exact values whenever suitable.*
☒	☐	For Bayesian analysis, information on the choice of priors and Markov chain Monte Carlo settings
☒	☐	For hierarchical and complex designs, identification of the appropriate level for tests and full reporting of outcomes
☒	☐	Estimates of effect sizes (e.g. Cohen's *d*, Pearson's *r*), indicating how they were calculated

Our web collection on statistics for biologists contains articles on many of the points above.

Software and code

Policy information about availability of computer code

Data collection	No software was used for data collection.
Data analysis	QuantSeq and RNASeq reads were mapped using the STAR and Cufflinks aligners, respectively. Proteomics data were analyzed using MaxQuant version 1.6.7.0. Differential expression analysis was performed using the PoissonSeq package in R. Gene Ontology Analysis was performed using PANTHER. Root length measurements were calculated using ImageJ. Statistical testing was performed using JMP Pro 15 and R (glmmPQL and phyper functions). Quantitative proteomics statistical analysis was performed using TMT-NEAT Analysis Pipeline version 1.4 (https://doi.org/10.5281/zenodo.5237316). TF-centered gene regulatory networks were inferred using SC-ION version 2.1 (https://doi.org/10.5281/zenodo.5237310).

For manuscripts utilizing custom algorithms or software that are central to the research but not yet described in published literature, software must be made available to editors and reviewers. We strongly encourage code deposition in a community repository (e.g. GitHub). See the Nature Research guidelines for submitting code & software for further information.

Data

Policy information about availability of data

All manuscripts must include a data availability statement. This statement should provide the following information, where applicable:

- Accession codes, unique identifiers, or web links for publicly available datasets
- A list of figures that have associated raw data
- A description of any restrictions on data availability

Raw sequencing data are deposited at the Gene Expression Omnibus with accession numbers GSE147589 [https://www.ncbi.nlm.nih.gov/geo/query/acc.cgi?acc=GSE147589] and GSE15700 [https://www.ncbi.nlm.nih.gov/geo/query/acc.cgi?acc=GSE157000]. Raw proteomics data have been deposited on MassIVE with accession number MSV000085606 [ftp://massive.ucsd.edu/MSV000085606/]. Source data are provided with this paper.

Field-specific reporting

Please select the one below that is the best fit for your research. If you are not sure, read the appropriate sections before making your selection.

☒ Life sciences ☐ Behavioural & social sciences ☐ Ecological, evolutionary & environmental sciences

For a reference copy of the document with all sections, see nature.com/documents/nr-reporting-summary-flat.pdf

Life sciences study design

All studies must disclose on these points even when the disclosure is negative.

Sample size	No calculations for sample size were performed. At least three biological replicates were used for multi-omics profiling and RT-qPCR experiments. At least 15 biological replicates were used for phenotyping experiments.
Data exclusions	Some QuantSeq samples were excluded due to poor read mapping and/or large variance from other biological replicates based on Principal Components Analysis. These samples were also excluded from the proteomics analysis for consistency across the data types.
Replication	At least three biological replicates were used for multi-omics profiling and RT-qPCR experiments. At least 15 biological replicates were used for phenotyping experiments. For RT-qPCR, two technical replicates were measured per biological replicate.
Randomization	Due to the nature of the experimental setup, randomization was not practical for this work.
Blinding	Due to the nature of the experimental setup, blinding was not practical for this work.

Reporting for specific materials, systems and methods

We require information from authors about some types of materials, experimental systems and methods used in many studies. Here, indicate whether each material, system or method listed is relevant to your study. If you are not sure if a list item applies to your research, read the appropriate section before selecting a response.

Materials & experimental systems

n/a	Involved in the study	
☐	☒	Antibodies
☒	☐	Eukaryotic cell lines
☒	☐	Palaeontology and archaeology
☒	☐	Animals and other organisms
☒	☐	Human research participants
☒	☐	Clinical data
☒	☐	Dual use research of concern

Methods

n/a	Involved in the study	
☒	☐	ChIP-seq
☒	☐	Flow cytometry
☒	☐	MRI-based neuroimaging

Antibodies

Antibodies used	anti-BES1: obtained from corresponding authors of reference #14: Yu, X. et al. A brassinosteroid transcriptional network revealed by genome-wide identification of BESI target genes in Arabidopsis thaliana. Plant J. 65, 634–646 (2011).<br>anti-Flag: obtained from Sigma-Aldrich (Cat #F7425, RRID: AB_439687)<br>anti-GFP: obtained from corresponding authors of reference #62: Yu, X. et al. Modulation of brassinosteroid-regulated gene expression by jumonji domain-containing proteins ELF6 and REF6 in Arabidopsis. Proc. Natl. Acad. Sci. 105, 7618–7623 (2008).
Validation	anti-BES1: validation performed in reference #14: Yu, X. et al. A brassinosteroid transcriptional network revealed by genome-wide identification of BESI target genes in Arabidopsis thaliana. Plant J. 65, 634–646 (2011).<br>anti-Flag: please see the manufacturer webpage: https://www.sigmaaldrich.com/US/en/product/sigma/f7425?context=product<br>anti-GFP: validation performed in reference 62: Yu, X. et al. Modulation of brassinosteroid-regulated gene expression by jumonji domain-containing proteins ELF6 and REF6 in Arabidopsis. Proc. Natl. Acad. Sci. 105, 7618–7623 (2008).
